# The Effect of Periodic Spatial Perturbations on the Emission Rates of Quantum Dots near Graphene Platforms

**DOI:** 10.3390/ma13163504

**Published:** 2020-08-08

**Authors:** Xin Miao, David J. Gosztola, Xuedan Ma, David Czaplewski, Liliana Stan, Haim Grebel

**Affiliations:** 1 Electronic Imaging Center and ECE Department, New Jersey Institute of technology (NJIT), Newark, NJ 07102, USA; xm37@njit.edu; 2 Center for Nanoscale Materials, Nanoscience and Technology Division, Argonne National Laboratory, Lemont, IL 60439, USA; gosztola@anl.gov (D.J.G.); xuedan.ma@anl.gov (X.M.); dczaplewski@anl.gov (D.C.); lstan@anl.gov (L.S.)

**Keywords:** semiconductor quantum dots, graphene, energy transfer, emission rate, emission lifetime

## Abstract

The quenching of fluorescence (FL) at the vicinity of conductive surfaces and, in particular, near a 2-D graphene layer has become an important biochemical sensing tool. The quenching is attributed to fast non-radiative energy transfer between a chromophore (here, a Quantum Dot, QD) and the lossy graphene layer. Increased emission rate is also observed when the QD is coupled to a resonator. Here, we combine the two effects in order to control the emission lifetime of the QD. In our case, the resonator was defined by an array of nano-holes in the oxide substrate underneath a graphene surface guide. At resonance, the surface mode of the emitted radiation is concentrated at the nano-holes. Thus, the radiation of QD at or near the holes is spatially correlated through the hole-array’s symmetry. We demonstrated an emission rate change by more than 50% as the sample was azimuthally rotated with respect to the polarization of the excitation laser. In addition to an electrical control, such control over the emission lifetime could be used to control Resonance Energy Transfer (RET) between two chromophores.

## 1. Introduction

The quenching of fluorescence in the vicinity of metals [1,2] and its extension to grapheme—a mono, or a few layers of graphite—has gained much interest lately for both biochemical sensing [3,4,5] and electronic applications [6,7,8,9,10,11]. If the potential barrier between the graphene and the quantum dots (QDs) does not allow for a direct charge transfer, energy may still be transferred via non-radiative dipole–dipole interaction [12,13,14]. Such Fluorescence Resonance Energy Transfer, (FRET), is enabled through the screening of the excited fluorophore by the free-carriers in the graphene film (a Förster process). For the energy transfer to be effective, the lifetime of a QD near the graphene needs to be shorter than the lifetime of a stand-alone QD. The absorption of graphene (~2.3% per layer) ought to be compatible with the absorption of the CdSe/ZnS QD monolayer so that the film of dots does not screen itself out (see comment [15]).

Intensity studies need to be complemented by time-resolved emission rates [16]. Concentration-dependent signals [17], masking the conductor by relatively thick QD films [18] and charge coupling between nearby dots may obscure the local interaction with the conductor.

Screening near the Dirac point by charged carriers depends on the amount of charge placed within a small distance away from the graphene [19]. Again, local laser intensities, local chromophore concentration and other scattering may affect fluorescence (FL) intensity variations. To a first-order, lifetime parameters are not affected by the laser intensity but are affected by the local density of states (DOS) [20,21,22,23]. The DOS for a 3-D system is proportional to the square of the radiation frequency. The DOS for a 2-D system is linearly proportional to the radiation frequency. Therefore, if energy is coupled to a 2-D guide before coupling to a 3-D free-space mode, the emission lifetime may be prolonged.

Our ultimate goal is to explore energy transfer from one QD to another via a surface guide as a mediator. To do so effectively, we first need to control the lifetime of the energy donating QD through a simple mechanism; in our case, this will be an azimuthal rotation of the substrate.

**The Coupling Process and its Theoretical Considerations:** the coupling process may be broken into several steps (see also Supplementary Information (SI) Section):

1. Excitation of the chromophore (here, the QDs) by a pump laser at frequency ω_L_. The chromophore is relaxed and transfers energy at frequency ω_E_ to a 2-D graphene surface guide on a hole-patterned oxide substrate. The graphene is coated with a thin oxide (hafnia). A surface mode is sustained due to the large refractive index of graphene (n_graphene_~2.6), QDs and hafnia, but not necessarily through a plasmonic mode for which the dielectric constant of graphene needs to be negative [24].
2. The excited QD dipole is coupled non-radiatively to a charge dipole in the graphene via energy transfer [9,10,11] at the rate of Γ_i1→f1_ with i1—the initial, excited state of QD and f1—the final state, the excited dipole in the graphene. The final state, f1 may transfer its energy to another QD nearby or thermally relax. If the graphene is coupled to a resonator (the periodic spatial pattern), then the QD may relax at a rate of Γ_i1→f2_ with i1—the initial, excited state of the QD and f2—the final electromagnetic state within the surface resonator. That mode may propagate back and forth along the surface resonator and eventually be coupled to free space modes or back to the lossy graphene film. Coherence in our case is achieved when the surface mode is at resonance with the local periodic perturbations.
3. A third interaction channel between the standing surface mode and the dipole generated in the graphene may be possible. Its mutual coupling may be sensitive to nonlinear photonic, or phononic effects [25] and could result in energy exchange. We will not dwell on such effect but a discussion is provided in the Supplementary information Section (SI). Overall, our measurements were carried for fluorescence intensity values that were linear with respect to the laser intensity.
4. The surface mode is coupled to free-space radiation modes and detected by a faraway detector.
5. Furthermore,

(a) when all the other parameters are kept the same, the emission rate of a chromophore coupled to a 2-D system is smaller than a chromophore coupled to a 3-D system;
(b) the conductive graphene increases the emission rate through non-radiative energy transfer process, which is enabled by charge screening;
(c) the effect of a resonating spatial perturbation is to further increase the emission rate of the chromophore due to an increase in the DOS near resonance [20]. The measured rate is Γ_ET_ = Γ_i1→f1_ + Γ_i1→f2_ in the absence of other nonlinear processes (see SI Section). The process efficiency is E~Γ_ET_/(Γ_ET_ + Γ_D_), where Γ_ET_ and Γ_D_ are, respectively, the non-radiative rate of energy transfer and the radiative decay rate of a stand-alone donor. Thus, increasing or decreasing of Γ_ET_ provides an active control over the entire process. Long lifetime donors (small Γ_D_) are preferred;
(d) the efficiency of the coupling between the QDs and the surface mode is enhanced because the resonating electromagnetic mode is mostly confined to the structure holes as we shall see below. A photon travelling back and forth within a resonating structure (namely, the surface guide with periodic perturbations) forms a standing wave at resonance conditions. The resonance conditions result in enhanced intensity at some particular tilt and azimuthal rotation angles with respect to the nano-hole array [16,26].

Following [27], once coupled to the surface guide, the mode propagates in the x-y plane with a wavevector, **β**_s_. A standing wave is formed if a Bragg condition is met: |**β**_s_**-G**| = β_s_; **G** is the reciprocal wave vector of the spatial square array of holes with a pitch. The wavenumber of the surface mode may be written as, β_s_~k_0_n_eff_ = (2π/λ_0_) × n_eff_. Here, λ_0_ is the free-space emission wavelength and n_eff_ is the effective refractive index of the surface mode (including the 10 nm hafnia, the QDs and their ligand coating).

An efficient coupling of the surface mode to and from free-space mode occurs if momentum is conserved: **β**_s_ = **k_o_**sinθ + q**G** with q integer (positive or negative). At normal incidence, we may pick up the x and y coordinate along the square hole-array coordinates, β_s_cos(φ) = q_1_G_x_ and β_s_sin(φ) = q_2_G_y_ with q_1,2_–integers and for square array, G_x_ = G_y_ = G (see SI Section). At normal incidence, coupling to the surface waveguide and the establishment of resonance conditions may occur simultaneously with the same angle φ and subwavelength patterns; for example if the scattering happens along the x-direction, m = 2q. A simplified numerical model is described in the SI Section.

The simulations indicate that the propagation along the x-direction in the surface guide may be polarized along either the y-direction (parallel to guide surface) or z-direction (perpendicular to the guide surface) for excitation and emission modes. For the emission wavelength, λ_0_ = 575 nm, resonance occurs at a normal direction, θ = 0° with φ = 0. For the incident wavelength and θ = 0°, resonance occurs at φ = 45° (along the cell’s diagonal) and the coupling to the surface guide is made with every other hole-plane q = 1/2. When excited by an s-polarizations (polarization parallel to the surface guide) there is a non-zero z-component (perpendicular to the guide surface) mostly in the air pillars. This implies that excited QDs, situated in, or nearby holes, are spatially correlated.

**Polarization and dephasing:** At normal incidence, the incident laser beam is polarized parallel to the guide’s surface. Simulations suggest that the pillar-interfaced, graphene surface guide supports Transverse Electric (TE) modes for the excitation and emitted wavelengths (the incident mode, E_y_, is parallel to the guiding surface). At normal incidence and through momentum conservation, this *s*-polarized excitation mode is coupled to two counter-propagating TE guided modes. The de-polarization [28] through relaxation of the excited carriers, from the excited state to the bottom of the conduction band, is small because this non-radiative process is very short (~100 fs) compared to the emission lifetime (~1 ns). Both the excitation and the emitted wavelength may be coupled via the hole-array. If, **β**_sL_, **β**_se_ are the surface wavevectors for the incident (laser) and emitted (fluorescence) modes, then, for a co-linear case, **β**_sL_ + **β**_se_ + **G** = 2π × n_eff_cos(φ)/λ_L_ + 2π × n_eff_/λ_e_ − 2π/*Λ* = 0 within 1% if we assume n_eff_ = 1.15 (see below). For a square array, we expect the transition rate to have a 90° rotation symmetry, considering two orthogonal Bragg reflectors along the x- and y-directions. Discussion on the fitting curve to the azimuthal data is provided in the SI Section.

## 2. Methods and Experiments

Since the energy transfer depends on the distance between the graphene and chromophore, a spacer may be used to control their mutual interaction. While very thin, this spacer—a 10 nm hafnia film on the graphene—in addition to the QDs and the graphene itself, may construct a surface optical waveguide. Coupling the excitation laser to this guide, or coupling the emission to free-space radiation modes may be conveniently made through a periodic array of nano-holes in the oxide substrate under the graphene layer. The array of holes provides for spatial confinement of the surface mode, effectively increasing its propagation lifetime but also increasing the emission rate of a nearby QD via an increase in the emission’s density of states (DOS) (Purcell effect).

All samples were made on a 500-micron-thick p-Si wafers coated with 150 nm SiO_2_. A square hole-array, with a pitch of 250 nm, a hole-diameter of 30 nm and a hole-depth of ca 30 nm was defined by e-beam lithography and etched into the SiO_2_ layer by using CHF_3_. The wafer was coated with ZEP diluted 1:4 anisole, spun at 4 krpm for 30 s and baked at 150 °C. A JEOL 8100 was used to write the pattern using 2 nA current and a dose of 300 μC/cm^2^. The sample was developed in n-amyl acetate for 60 s, rinsed in Isopropyl Alcohol (IPA) for 30 s and then dried with N_2_. The devices were etched for 2 min in DC reactive ion etching (50 sccm CHF_3_ 2 sccm O_2_) 50 W power at 10 mTorr

A monolayer graphene was grown by the Chmical Vapor Deposition (CVD) method as described in [29]. The graphene was coated with 10 nm hafnia by atomic layer deposition (ALD) prior to the graphene transfer as per our recipe described elsewhere [30]. In short, the graphene transfer process involves coating the hafnia-covered graphene with an additional, 250 nm layer of poly(methyl methacrylate), PMMA, which can be retained as an optical waveguide or may be easily dissolved, leaving the hafnia-covered graphene intact. Note that the ALD is made at a relatively high-temperature of ca 200 °C, which may prohibit its use if the QDs are already placed in the hole-array.

**Figure 1:**
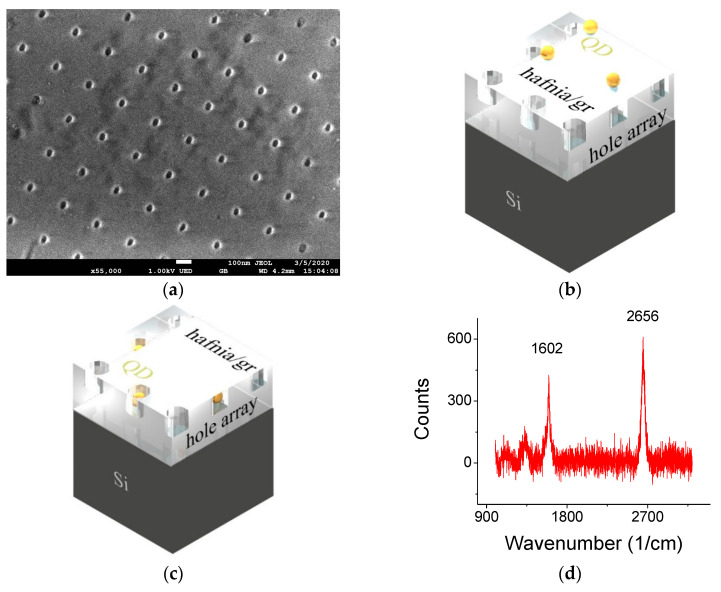

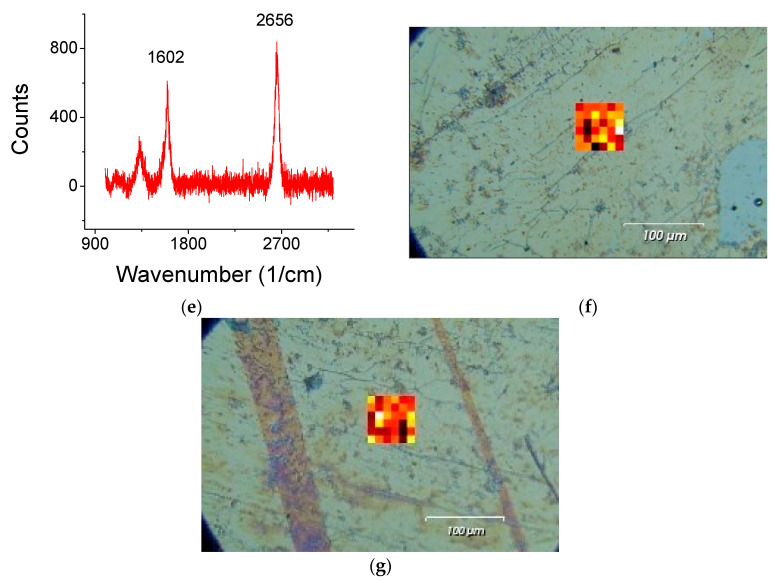
(**a**) Scanning Electron Microscope (SEM) picture of the bare substrate with a hole array. (**b**,**c**) Schematics of samples S9 (spun QD on top of the graphene/hafnia surface guide) and S8 (spun and wiped QD under the graphene/hafnia guide). (**c**) Typical Raman spectra taken with a 2 mW, 633 nm HeNe laser; (**d**) was obtained when the dots were deposited on top of the hafnia/graphene layer, while (**e**) was obtained when the dots were deposited under the graphene. (**f**,**g**) Raman maps of the graphene’s 2D line for (**f**) top and (**g**) under deposited dots allude to the monolayer nature of the graphene. The 2D intensity values, I_2D_, for the black, red and white squares were, 500, 1000 and 3000, respectively. The ratio of I_2D_ to the intensity of the G line, I_G_ was approximately I_2D_/I_G_ = 1.3 throughout the scan.

The QDs (core/shell, CdSe/ZnS [31]) were purchased from Mesolight (Suzhou, China) and were deposited either on top of the hafnia/graphene layer (Figure 1a) or underneath it within the holes (Figure 1b). For the latter case, special attention was given to maintain as many dots inside the holes and remove excess dots from the oxide surface (where direct contact is made with the graphene). When the QDs are embedded in the holes, the filled holes may accommodate only one dot per hole since the dot is coated with a ligand whose overall diameter is ca 20 nm. The dots are situated at the hole’s bottom due to capillary action [16] and the separation between the dot and the graphene would be 20 nm (the hole’s depth minus the radius of the ligand coated dot). When the dots are deposited on the top of the 10 nm hafnia, the separation between the dot and the graphene can be more accurately maintained, being 20 nm, as well. As described in Table 1, when the QDs are on top of the graphene, their distance from one another is random. When the dots are below the graphene and inside the hole-array, they are well separated and coupled by hole-array symmetry, with high and low QD concentrations.

Raman spectra were taken with a 633 nm HeNe laser (above the excitation and emission wavelengths of the QD) at an intensity of 2 mW as an excitation source and a × 20 objective. Stresses in the graphene from the hafnia and the QDs may affect its 2-D line, albeit its position remained in the vicinity of 2650 1/cm. The QD emitting in the wavelength of ca 575 nm were pumped with a 90 ps, 250 μW, 405 nm laser at a pulse rate of 25 MHz. The fluence was 1000 W/cm^2^. The dots, suspended in toluene were either dip-coated or spun at 2500 RPM for 30 s.

For the time-resolved and fluoresce measurements, the laser beam at 405 nm was focused by an × 5 objective to a ca 25 micron^2^ spot onto a well-defined spot that was visible through the set of filters and could be visited time and again. The sample area could be viewed using a home built optical microscope that could be separated from the measurement system by a prism and which was equipped with a white light illuminator and a Charge Coupled Device (CCD) camera while viewing through the same objective. An error in repeating the measurement of the same spot was estimated as less than 10%. Uncertainties in the exact spot position may have contributed to coefficient variations. For the fluorescence data, the detector was equipped with a cut-off low-pass filter whose cut-off wavelength was 450 nm. For the time-resolved measurements, a bandpass filter between 500 and 700 nm was used (with a different detector than the one used for the fluorescence measurements).

The FL curves exhibited multiple decay rates and could be properly fitted with three decay constants. In each case, the entire curve (from the stating pulse at ca 6 ns to 200 ns) was used for fitting. The largest decay rate is of the order of 2 ns^−1^. It is attributed to dots that are in close proximity to the graphene guide. The medium rate is of the order of 0.2 ns^−1^ and is attributed to dots that are less impacted by the graphene layer. The smallest decay rate is of the order of 0.05 ns^−1^ and serves as a background component and could be also attributed to the photon lifetime in the waveguide. As the focal point moves away from the graphene surface, the weight of the three ensembles is shifted towards dots that are less impacted by the graphene surface.

Here are some considerations to the fit process that led to the evaluation of the emission rates.

(1). The geometrical effect of the laser spot on the overall emission rate assessment is not straight forward. The Gaussian beam has features of a plane wave only at the focal point; yet, excitations of the dots with varying degrees of efficiency occur with the unfocused beam as well.
(2). Limiting the fit to mainly one time component that is prevalent in a finite time range (namely, limiting the fit, say, to a window of 10 ns after excitation) runs the risk that the solution will be affected by the boundaries of the time window.
(3). Having too many time constants may blur the physics of the processes.
(4). Considering the fit quality by only its R-square value is insufficient. One needs to consider the distribution of the residuals about the fitting parameter (see SI Section). The residual distribution has to be evenly spread above and below the mean.

## 3. Results and Discussions

**Figure 2:**
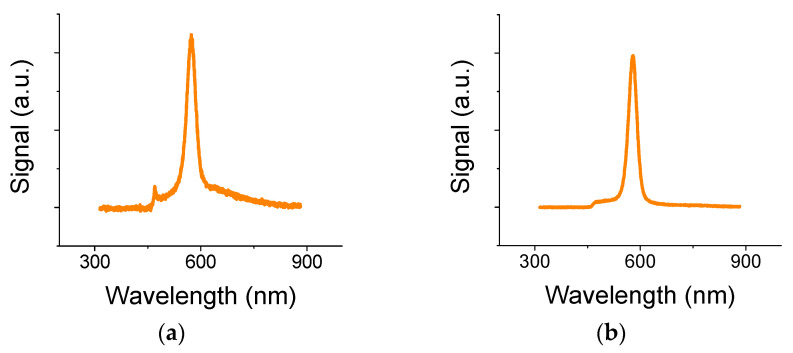
Full fluorescence curves for (**a**) sample S7 with 10 nm hafnia and (**b**) sample S2 without it. A laser cutoff filter was placed at 450 nm.

In Table 1, we provide a description of four samples that were prepared in various ways. Common to them is the average spacing between the QD and graphene (either when the QD are above the graphene and randomly dispersed, or when they are below it, embedded in the holes, well separated and their fluorescence is coupled by the surface mode wavevector). A Scanning Electron Microscope picture of a bare patterned substrate is shown in Figure 2a. A detailed description of the samples is provided in Figure 2b,c and Table 1. Typical Raman spectra taken when the dots were deposited on top of the hafnia/graphene layer, or deposited under the graphene (while still with the hafnia on top) are shown in Figure 1d,e. Raman maps of the 2D line for the two cases are shown in Figure 1f,g, respectively, and point to the monolayer nature of the graphene throughout the sample. While the scan was taken from a limited area of 60 × 60 μm^2^, the graphene film was much larger, approximately 5 × 5 mm^2^. The maps are overlaid on an image of the substrate; some cracks formed in the hafnia during the transfer of the hafnia/graphene film are noted.

A typical full fluorescence (FL) curve for sample S7 (with 10-hafnia layer on top of the graphene) exhibits a 470 nm line that is attributed to color centers of the 10 nm hafnia on graphene (Figure 2a). The line is missing from sample S2 that lacks the hafnia layer (Figure 2b), yet coated with a 250-nm-thick PMMA on top of the graphene acting as a surface waveguide. The time-resolved curves, shown below, were obtained with a bandpass filter between 500 and 700 nm.

**Figure 3:**
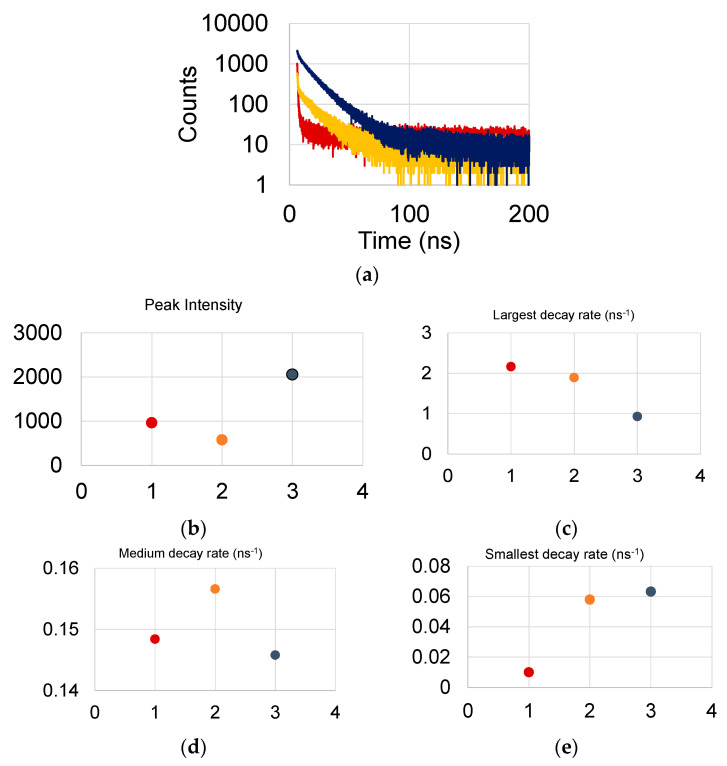
(**a**) Red to blue: as the focus of the laser beam is receding away from the sample surface, (**b**) the peak intensity changes, (**c**) the larger decay rate becomes smaller, (**d**) the medium decay rate remains fairly constant and (**e**) the smallest decay rate increases.

The effect of focusing the laser beam on the measured time rates is shown in Figure 3 for sample S9 where the QD were spun over the hafnia/graphene layer. Three curves are shown for which the focal point was successively receding away from the sample surface. The peak intensity of the curve substantially varied for these three cases. This could be the result of: (a) the laser interrogated QDs that are at various distances from the quenching graphene layer, or that (b) the ach focal point interrogated different QD ensembles.

**Figure 4:**
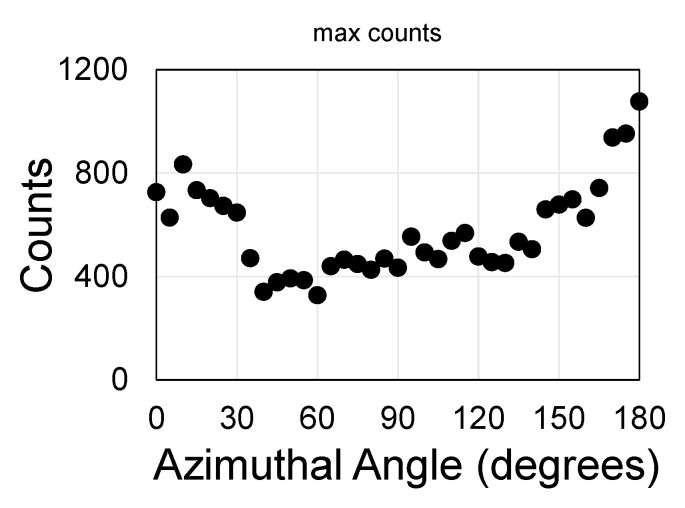
Variations of the peak intensity as a function of azimuthal angle, φ between the laser polarization and the hole-array orientation do not reveal a 90° symmetry; the 180° symmetry alludes to the repeatability of the rotation experiments.

A more detailed description is provided below for sample S7. The QDs were deposited on the hafnia/graphene layer by use of dip-coating. Plotting the maximum count vs. azimuthal angle, φ, between the laser polarization and the hole-array orientation yields mainly two peaks, at 0° and at 180°, that allude to the stability and repeatability of the measurements (Figure 4) but do not clearly exhibit much symmetry that can be related to the square hole-array.

**Figure 5:**
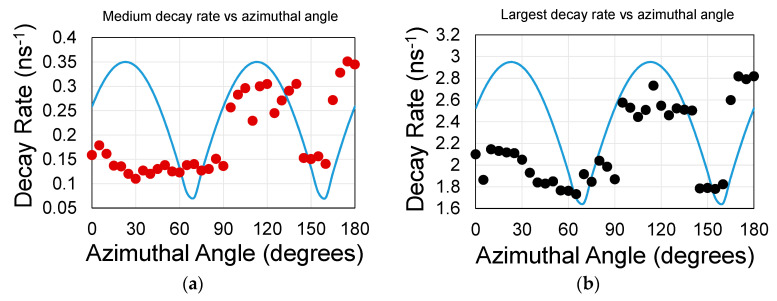
Sample S7. There are essentially two decay rate coefficients: (**a**) below and (**b**) above 1 ns^−1^. The blue lines in (**a**,**b**) are guide for the eyes (see SI Section). The curve was shifted by φ_0_ since the initial hole-array orientation was unknown. The error in the decay rate fit was less than 1% (and hence is contained within a data point); the error in the azimuthal angle was 0.5°. The error in repeating the measurement of the same spot is less than 10%. In general, QDs, which are deposited on top of the graphene, exhibit clearer undulations.

We concentrate on the first two major decay rates: the largest (of ca 2 ns^−1^) and the medium (of ca 0.2 ns^−1^) rates. The medium decay rate distribution as a function of the azimuthal rotation angle φ is shown in Figure 5a. The largest decay rate is shown in Figure 5b. The medium decay rate is equivalent to an average life time constant of 6 ns. That value is within an order of magnitude for a stand-alone QD (on a 10 ns scale). The largest decay rate is equivalent to a lifetime constant of 0.5 ns. It exhibits a more pronounced 90° cycle symmetry as indicated by the blue line and as expected by the square nano-hole symmetry. The blue line also indicates that the coupling constant, κ, and the interaction length between surface mode and the hole-array planes behave as, κL~1.

The error in the decay rate fit is less than 1% (and hence is contained within a data point); the error in the azimuthal angle is 0.5°. We attempted to maintain the same spot position during sample rotation. The error of maintaining that spot is estimated at less than 10%. Yet, uncertainties in the exact spot position may have contributed to coefficient variations.

**Figure 6:**
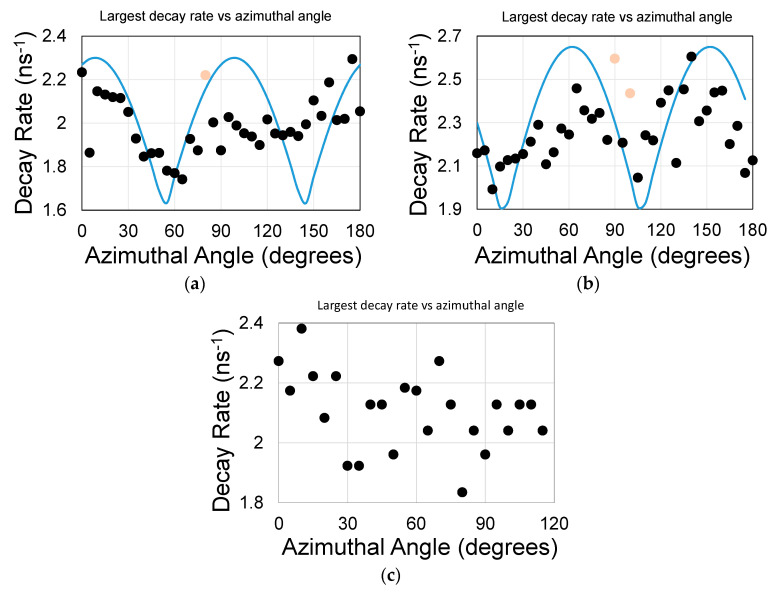
(**a**) Sample S8 (QDs in the holes under the graphene guide) and (**b**) sample S9 (QDs on top of the 10-nm hafnia on the graphene guide). The QDs were spun over the samples. The blue line is the expected undulations and a guide for the eyes. The red dots indicate outliers: a solution was achieved with a good R square value of above 0.97 but residuals were not evenly distributed about the mean (see SI Section). (**c**) Maximizing the fluorescence signal (as opposed to focusing onto the sample surface) resulted in decay rates that covered similar value range to (**b**) but failed to uncover meaningful undulations.

The largest decay rate coefficient as a function of azimuthal angle φ for samples S8 (QD under the graphene) and S9 (QD on top of graphene) are shown in Figure 6a,b (see also SI Section). Unlike sample S7, here the QD were spun over the surface and their concentration was less than S7 (25% of S7 concentration). The data undulations are more pronounced for QD placed on top of the graphene, yet ‘cleaner’ for QD deposited underneath it. Similar emission rates for top-coated and under-coated QDs are the result of similar distance from the graphene layer. The 90° symmetry is consistent with n_eff_ = 1.15. Figure 6c shows data for sample S9 when keeping the same spot position and maximizing the FL intensity (as opposed to focusing the spot onto the sample surface). The range of the larger decay rate is similar to the overall range of Figure 6b, yet without an apparent undulations.

**Figure 7:**
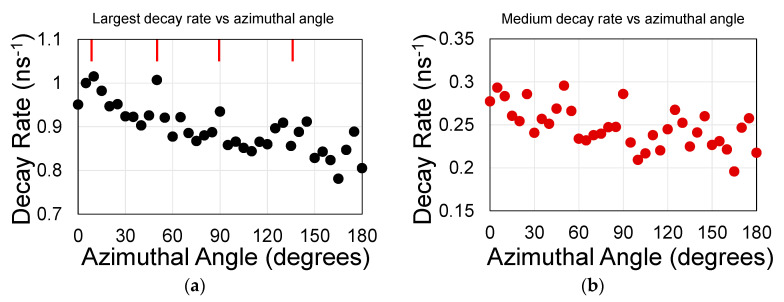
Decay rate coefficients in ns^−1^ for sample S2 exhibiting 45° symmetry for both, (**a**) the largest rate and (**b**) the medium rate. The red lines point to the peaks.

If surface guide is interfaced with a relatively top thick polymer instead of air the surface guide becomes more symmetric. The 250 nm PMMA layer, used during the transfer stage of graphene was retained and no oxide was deposited on top of the graphene. Sample S2 was made of spun QD in the nano-hole array and under the graphene layer. Judged by the emission rates, the dots resides away from the graphene. As exhibited by Figure 7, and unlike the previous samples, both the largest and medium decay rate coefficients exhibited 45° undulations. This was made with the (½, 0) planes, or every other plane. The Bragg peaks appear every 45° and the reflectivity is much narrower than for an air-top guide (SI Section).

There are a few more questions that need to be addressed:

What is the role of the graphene thickness? As the graphene thickness increases so does its conductivity and thus its screening effect. If the QD are placed above the surface guide, their interaction with the hole-array would be decreased. An increase in the graphene thickness will also increase its electromagnetic losses. The effect of multilayer graphene on the lifetime of the QDs was studied by us [16] and others [32] in the past.

What is the effect of temperature? It has been shown that the specific conductance of graphene decreases with an increase in temperature in the range 0–180 K and increases with an increase in temperature in the range 180–800 K [33,34]. This means that the screening effect would increase for relatively high temperatures and the emission rate would increase as well.

Finally, what about QDs at longer wavelengths? The effects described here are scalable up to λ~10 μm when the graphene becomes plasmonics (namely, portrays a large negative refractive index). Beyond that wavelength, the surface guide becomes much more effective [25] and higher orders of nonlinearity may be observed. One potential application would be efficient mid-IR sensing devices.

## 4. Conclusions

We observed variations in the quenched fluorescence’s lifetime of QDs embedded with patterned quasi two-dimensional graphene surface guides upon azimuthal rotations. These variations were as large as 50%. Since coupling to spatially resonating surface modes is also associated with large emission rates for nearby chromophore, one, in principle, could control an energy transfer from one type of dot to another via the graphene surface guide. In addition to an electrical control [7,35], spatial perturbation may not only control the chromophore emission rate but also enable an efficient biosensing tool.

## Figures and Tables

**Table 1:** Sample description. Spinning was made at 2500 RPM for 30 sec; the quantum dot (QD) concentration was: High = 1 mg/mL; Low 0.25 mg/mL; dipping was made with a high concentration at a speed of 2 mm per minute; the spacer is the 10 nm hafnia on top of the graphene.

Sample	QD Deposition Method	Placement of QD	Concentration	Spacer/Top Coat
S2	spin	in holes	High	no/PMMA
S7	dip	on spacer	High	yes/no
S8	spin	in holes	Low	yes/no
S9	spin	on spacer	Low	yes/no

## Supplementary Materials

The following are available online at https://www.mdpi.com/1996-1944/13/16/3504/s1, Figure S1: Schematics of the QD coupled to graphene and a surface resonator; Figure S2: An s-polarized incident beam forming two counter propagating Transverse Electric (TE) waveguide modes. The polarization (black arrows) is preserved due to fast non-radiative life-time. The waveguide supports both TE and Transverse Magnetic (TM) modes; Figure S3: (a) A plane wave is excited from the left (x = 0) and allowed to propagate in all directions. A portion of the wave is captured by the surface guide. Shown is E_y_ (parallel to the guide surface) at the effective surface guide between guide and silica film. Note the focusing of the beam by the sub-wavelength hole-array. (b) E_z_ (perpendicular to the guide surface) at the effective surface guide between guide and silica film. (c) E_y_ for a spherical wave excited by a QD in one of the holes on the left and is let to propagate along all directions. The surface guide is made of air–guide–silica layers. (d) E_z_ at the guide–silica interface for a spherical wave when propagating in a guide made of air–guide–silica layers. The emission wavelength was 575 nm and the intensity legends are in V/m; Figure S4: (a) Superimposed curves of Bragg scatterings (red and blue curves) corresponding to the scattering by the x- and y-planes of the hole-array for κL~1 when the surface guide is (a) topped by air and (b) topped by a polymer. For (a), the scatterings were made by the (10) and (01) planes. We used a simplified approximation ~|cos(2ϕ)| (magenta) to accentuate the trend. For (b), the scatterings could be made by the (½,0) and (0,½) planes (as shown) for every other plane or alternatively by the (1,0) and (1,1) planes. The latter condition requires a much large coupling constant, though κL~3. The peaks in (b) are clearly narrower, corroborating Figure 7 in the text; Figure S5: (a) A good fit with proper residuals (a,b). (a) Bare oxide outside the hole-region. (b) Inside the hole-region—the time constants have been substantially reduced for QDs situated inside the hole-region. (c) S9 outlier at ϕ = 100°. The R-square = 0.95, yet the residuals are not distributed evenly and tilted towards the positive part. In addition, the fitting curve starts at 3.2 ns, down shifted from ca 6 ns, the starting point for the experiments; Figure S6: (a) S8 (QD below the graphene inside the holes) and (b): for S9 on top of the hafnia above the graphene layer. The gray dots are outliers.
