# Supplementary material for: The Effect of Periodic Spatial Perturbations on the Emission Rates of Quantum Dots near Graphene Platforms
Source: Materials (Basel). 2020 Aug 8;13(16):3504. doi: 10.3390/ma13163504 (PMC7475916; doi:10.3390/ma13163504)
Supplement: Supplementary file 1 [file materials-13-03504-s001.zip › materials-848982-supplementary.pdf]

materials

MDPI

Supplementary Materials

The Effect of Periodic Spatial Perturbations on the Emission Rates of Quantum Dots near Graphene Platforms

Rate Equations:

The system is pictorially presented in Figure S1:

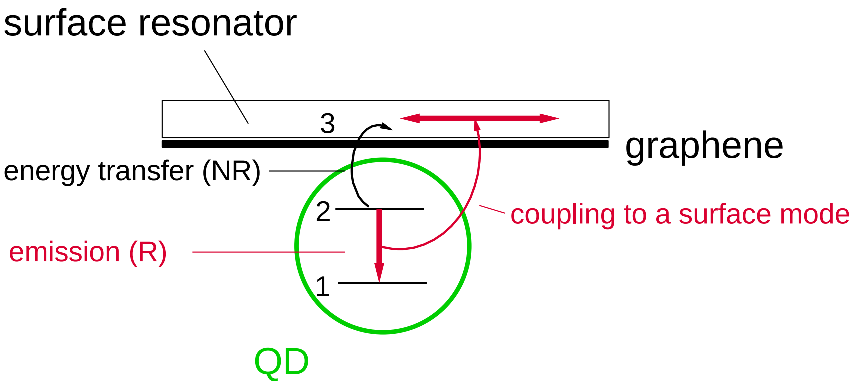

Figure S1. Schematics of the QD coupled to graphene and a surface resonator.

We assume a three-level system. A pulse R ($t < 0$) excites the QD to level 2 from level 1. The emission may be coupled non-radiatively (NR) to level 3 at time τ_{23}; it may emit a photon at time τ_2 and couple radiatively (R) to the resonating surface mode. The radiative emission rate, $\Gamma_{2\to 1}$ = $1/\tau_{21}$~$\varrho(\upsilon_{21})H_{21}$ includes the interaction term H_{21} and the density of the final state, $\varrho(\upsilon_{21})$ (Fermi's golden rule) [1]. The density of states (DOS) for a resonating mode is basically 1 mode per mode's width $\Delta\upsilon$ per volume V or $1/(\Delta\upsilon \times V)$. The mode spectral width is written as: $\Delta\upsilon^{-1} = Q/\upsilon_{21}$, with Q, the resonator's quality factor, and υ_{21}, the transition frequency; the mode volume is: V~$(p \times \lambda/2)^2$, where p is the characteristic decay length away from the surface and $\lambda_{21} = (c/n)/\upsilon_{21}$ is the mode wavelength. The characteristic decay length of the mode, p, is a fraction of a wavelength, typically, $\lambda/4$ near conductive surfaces. Thus, for a surface resonator, the density of states is increased by the quality factor Q compared to a free-space QD and consequently, the spontaneous emission rate is increased by Q, as well (Purcell's effect) [2,3]. We assume that when the transition frequency coincides with the resonator mode, the transition rate is dominated by the DOS of the resonator.

We use the following rate equations for a pulse pump, R:

$$dn_2/dt = -n_2/\tau_{21} - n_2/\tau_{23} - \sigma n_2 n_{ph} + R(t<0) \quad \text{(S1)}$$

$$dn_3/dt = -n_3/\tau_{33} + n_2/\tau_{23} + \sigma n_3 n_{ph} \quad \text{(S2)}$$

$$dn_{ph}/dt = -n_{ph}/\tau_{ph} + \sigma n_2 n_{ph} - \sigma n_3 n_{ph} \quad \text{(S3)}$$

Here: n_2 is the excited electron density, n_3 is the excess electron density in the graphene due to non-radiative energy transfer, n_{ph} is the output photon density, τ_{21} is the life-time of the radiative emission (coupled to the surface resonator), τ_{23} is the non-radiative transition to the graphene, τ_{33} is the dissipation time and τ_{ph} is the photon life-time in the surface resonator. The cross-section, σ is assumed to be equal for the lossy photons; τ_{ph} is rather short.

The solution of Equation S1, is $n_2(t) = n_2(0)e^{-t/\tau_{2eff}}$, with $1/\tau_{2eff} = 1/\tau_{21} + 1/\tau_{23} + \sigma n_{ph}$; the stand-alone radiative emission rate of the QD, $1/\tau_{21}$ is increased by the non-radiative transfer of energy to the graphene and the presence of increase mode density in the resonator.

Let us assume that the presence of n_3 is only due to n_2, $n_3 = \alpha n_2$.

The excess charge in the graphene is $n_3(t) = n_3(0)e^{-t/\tau_{3eff}}$, with $1/\tau_{3eff} = 1/\tau_{33} - 1/\alpha\tau_{23} - \sigma n_{ph}$. Interestingly, the rate of exchange energy (negative sign for a gain), $-1/\alpha\tau_{23}$ is accentuated by the weak coupling between the QD and graphene ($\alpha \ll 1$). Weak coupling alludes to larger distances between the QD and graphene and the interaction term H_{23} that enables the coupling substantially decreases as a function of distance [4].

Numerical Assessments:

The coupling to surface modes is pictorially shown in Figure S2.

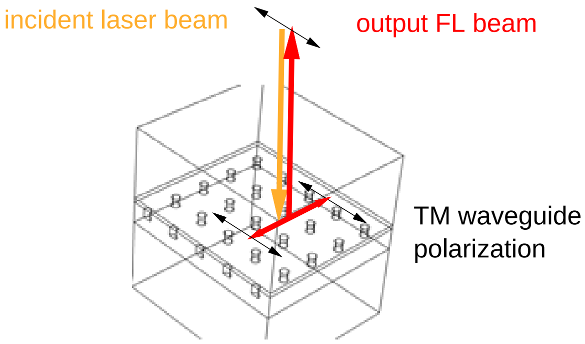

Figure S2. An s-polarized incident beam forming two counter propagating Transverse Electric (TE) waveguide modes. The polarization (black arrows) is preserved due to fast non-radiative life-time. The waveguide supports both TE and Transverse Magnetic (TM) modes.

The various electric field distributions are evaluated at a cut-plane at the interface between the waveguide and the substrate (Figure S3). The model was constructed with a CAD tool (COMSOL). Scattering boundaries were used around the structure. These are equivalent to perfectly matched layers (PML) to avoid back reflections. A thick Si wafer (bottom) is covered by a 150 nm silica film, which is decorated with air pillars of depth 50 nm. The pillars of radius 30 nm are covered with a surface guide and are topped either by air, or a polymer film with a refractive index similar to that of the silica. The pitch of the hole-array is 250 nm. The surface waveguide is composed of graphene, 10 nm hafnia and QDs. The QDs (with a radius of ca 3–4 nm) and their ligand coating have an effective thickness of 20 nm. The effective thickness of the guide is 30 nm and its refractive index is 2.4 + i0.24. As we shall see below, the actual refractive index of the optical surface guide is of little consequence because most of the mode intensity propagates outside it. In Figure S3, we show two cases: (1) a collection of many dots that form a plane wave at the emission wavelength of 575 nm along the x-direction and polarization along the y-direction (the waveguide's TE mode) and (2) an emission from a single trapped dot as a spherical wave. The electric fields, polarized along the y- and z-directions were assessed.

(1) A plane wave of 1 V/m and whose polarization is along the y-direction (parallel to the guide surface) propagates along the x-y plane of the surface guide. Figure S3a shows the E_y component (parallel to the guide surface) and Figure S3b is for Ez polarization (perpendicular to the guide surface). The effective index of the surface guide can be deduced when referencing the wavelength along the interface to the array pitch of 250 nm. Thus, $\lambda_n = \lambda_0/n_{eff} \sim 2a$ for the air-topped sample fulfilling the Bragg condition along the x- and y- directions. This is translated to $n_{eff} \sim 1.15$, which is consistent with the experiments. Similarly, for a guide surrounded by polymer and silica, $n_{eff} \sim 1.45$. Both cases allude to the fact that the wave travels mostly outside the surface waveguide. Interestingly, if the average permittivity of the air/quartz interface, $n^2_{eff} = \varepsilon_{eff} = (\varepsilon_{air} + \varepsilon_{silica})/2 = (n^2_{air} + n^2_{silica})/2$, or $n_{eff} = 1.25$.

(2) The case where the waveguide is excited by a spherical point source (QD), which is situated in one of the holes is presented in Figure S3c,d. The wavelength match is $\lambda_0/(n_{eff} = 1.15)$~2a and a higher orders, for air topped and polymer topped, respectively. Another intuitive view is to consider the graphene/hafnia/OD interface as either asymmetric guide when the bottom layer is made of silica and the top layer is air, or, a symmetric guide when the top layer is made of a polymer. In either case, most of the surface mode is propagating outside the guide and simulations imply that the propagation is above waveguide cut-off. Finally, Figure S3d shows the Ez polarization component (perpendicular to the guide surface) when excited by a spherical wave. The component is not zero and is concentrated in the pillars.

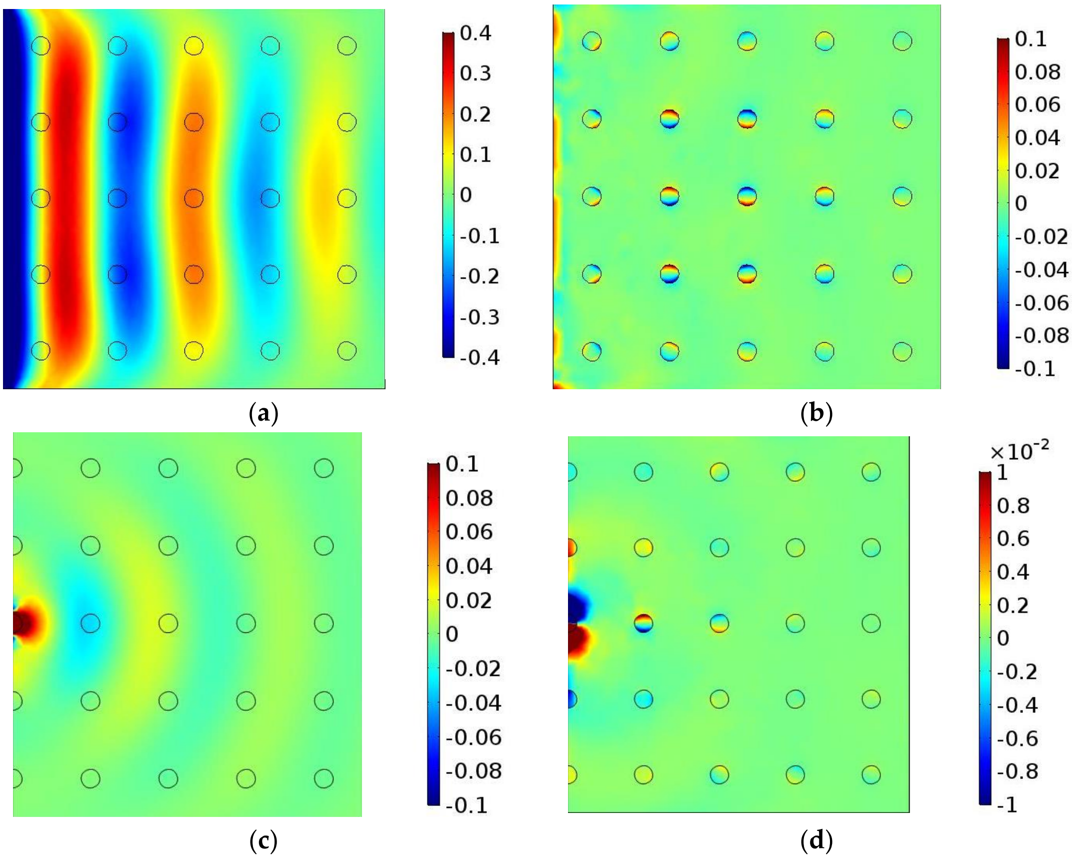

Figure S3. (**a**) A plane wave is excited from the left (x = 0) and allowed to propagate in all directions. A portion of the wave is captured by the surface guide. Shown is E_y (parallel to the guide surface) at the effective surface guide between guide and silica film. Note the focusing of the beam by the sub-wavelength hole-array. (**b**) E_z (perpendicular to the guide surface) at the effective surface guide between guide and silica film. (**c**) E_y for a spherical wave excited by a QD in one of the holes on the left and is let to propagate along all directions. The surface guide is made of air–guide–silica layers. (**d**) E_z at the guide–silica interface for a spherical wave when propagating in a guide made of air–guide–silica layers. The emission wavelength was 575 nm and the intensity legends are in V/m.

At normal incidence, we may pick up the x- and y- along the square hole-array coordinates. **G** is the reciprocal wave vector of the spatial square array of holes with a pitch Λ; $G_x = G_y = G$. Coupling to and from the hole-array at a normal incident fulfils $\beta_s = G[q_2^2 + q_2^2]^{1/2}$with $q_{1,2}$, integers, and $\beta_s = 2\pi\lambda/n_{eff}$, the wavenumber of the surface mode. The Bragg condition is $|\boldsymbol{\beta}_s - \mathbf{G}| = \beta_s$. Thus, $2\Lambda\cos\phi = m\lambda/n_{eff}$. At a normal incidence, we may pick up the x- and y- along the square hole-array once coupled to the surface mode, we can approximate the in-plane reflections as (counter propagating coupled mode theory [5]),

$$R = \frac{\kappa^2 sh^2(s \times L)}{s^2 ch^2(s \times L) + (\frac{\Delta\beta_s}{2})^2 sh^2(s \times L)} \qquad \text{(S4)}$$

where, $s^2 = \kappa^2 - (\frac{\Delta\beta_s}{2})^2$, κ, is the coupling constant between the hole-array planes and the propagating guided mode, and $\Delta\beta_s = 2k\cos(\Phi) - qG$ and L is the effective interaction length. Figure S4 shows a super imposed curve for the Bragg scatterings from the x- and y-directions. For κL~1, the curve can be simply approximated by │cos(2ϕ)│ (magenta curve) and was used to accentuate the azimuthal curves.

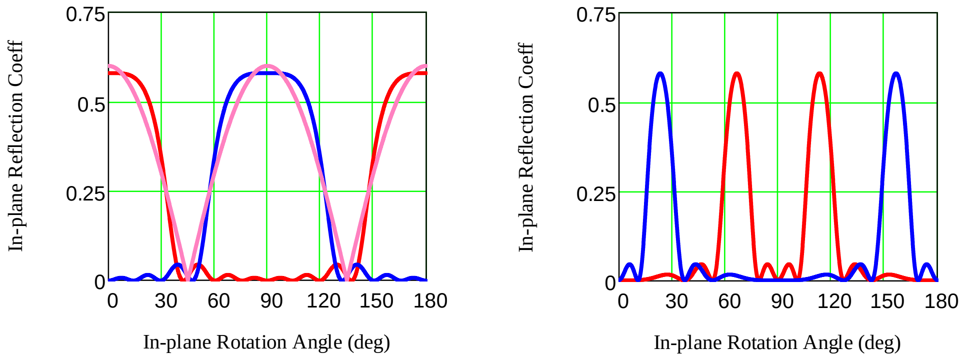

(**a**) (**b**)

Figure S4. (**a**) Superimposed curves of Bragg scatterings (red and blue curves) corresponding to the scattering by the x- and y-planes of the hole-array for κL~1 when the surface guide is (**a**) topped by air and (**b**) topped by a polymer. For (**a**), the scatterings were made by the (10) and (01) planes. We used a simplified approximation ~│cos(2ϕ)│ (magenta) to accentuate the trend. For (**b**), the scatterings could be made by the (½,0) and (0,½) planes (as shown) for every other plane or alternatively by the (1,0) and (1,1) planes. The latter condition requires a much large coupling constant, though κL~3. The peaks in (**b**) are clearly narrower, corroborating Figure 7 in the text.

In Figure S5a,b, we provide curve fitting examples for reference points on the oxide without graphene for sample S9: (a) outside the hole-array region and (b) inside it. Time constants for the inside the hole-region have been reduced. The relevant rate (the larger decay rate) has been increased from ~1 ns^{-1} outside the hole-region to1.6 ns^{-1} inside it. As discussed in the text, a good fit ought to consider not only its convergence but the distribution of its related residuals. As shown in Figure S5c, the point for sample S9 at ϕ = 100° is considered an outlier because the residuals are not evenly distributed above and below the zero-line (in other words, the data points are not completely random). In addition, the fitting curve starts at 3.2 ns, down shifted from ca 6 ns, the starting point for the experiment.

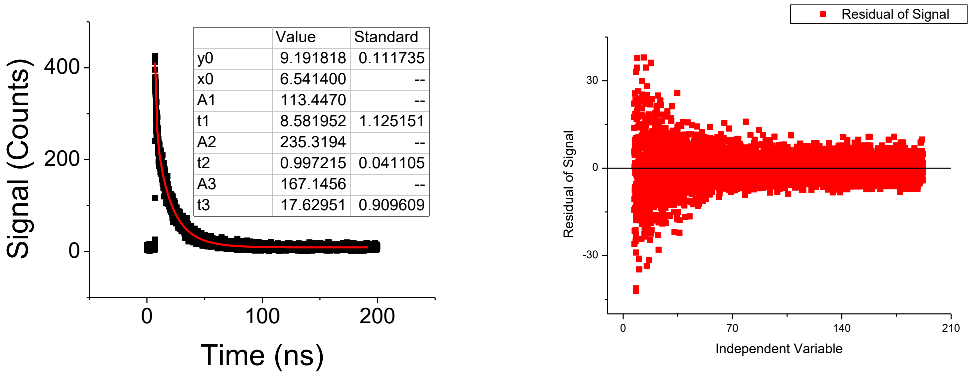

(**a**)

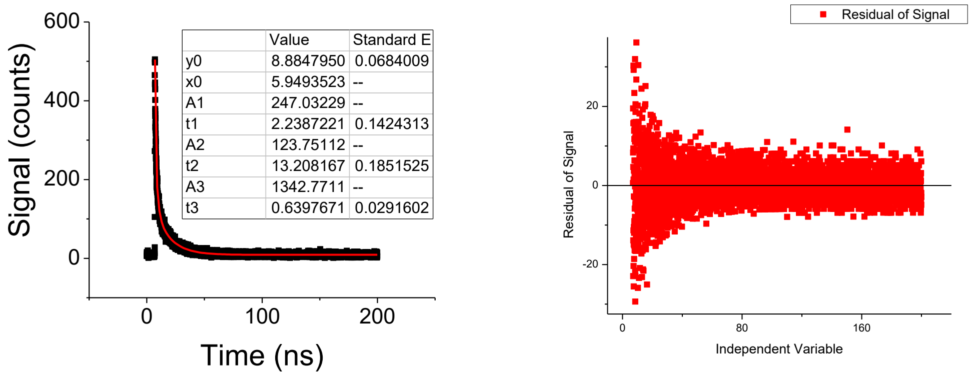

(**b**)

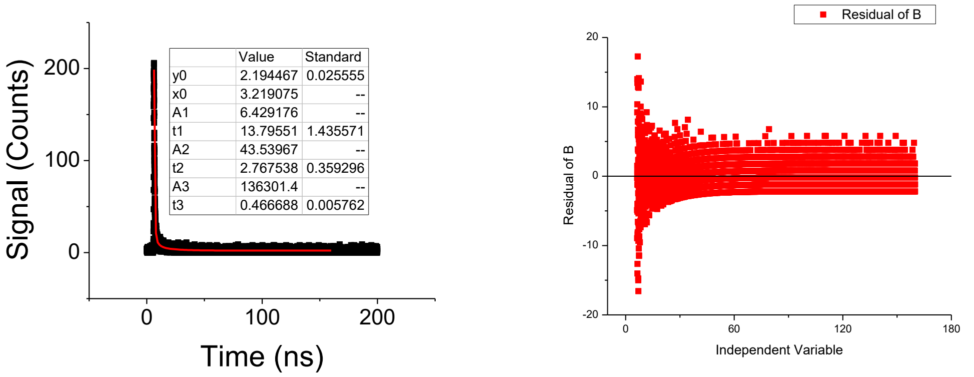

(**c**)

Figure S5. (**a**) A good fit with proper residuals (**a**,**b**). (**a**) Bare oxide outside the hole-region. (**b**) Inside the hole-region—the time constants have been substantially reduced for QDs situated inside the hole-region. (**c**) S9 outlier at $\phi = 100°$. The R-square = 0.95, yet the residuals are not distributed evenly and tilted towards the positive part. In addition, the fitting curve starts at 3.2 ns, down shifted from ca 6 ns, the starting point for the experiments.

Figure S6 shows the 'medium' decay rate for samples S8 (QD below the graphene inside the holes) and for S9 on top of the hafnia above the graphene layer. The undulations are not as clear as for the 'largest' decay rates.

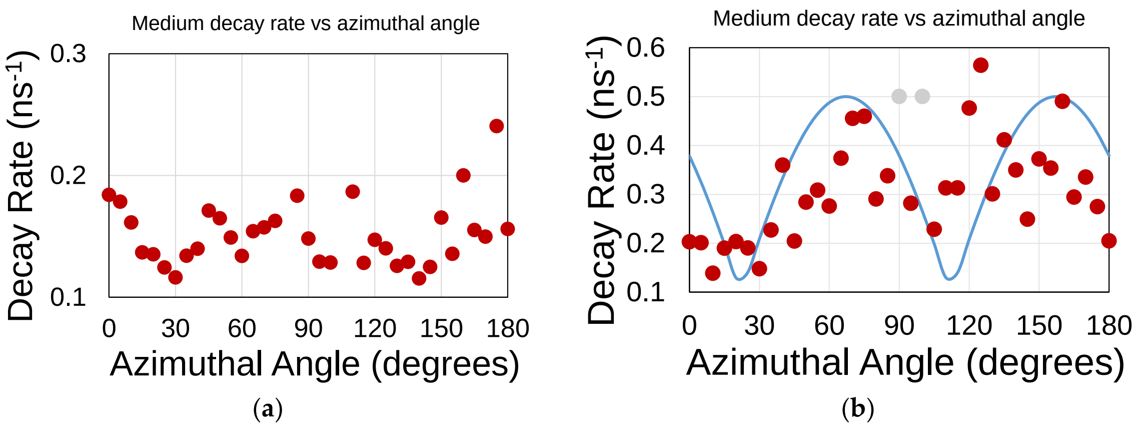

Figure S6. (**a**) S8 (QD below the graphene inside the holes) and (**b**): for S9 on top of the hafnia above the graphene layer. The gray dots are outliers.

Reference

1. Peyghambarian, N.; Koch, S.; Mysyrowicz, A. *Introduction to Semiconductor Optics*; Prentice Hall: Englewood Cliffs, NJ, USA, 1993
2. Purcell, E.M. Spontaneous emission probabilities at radio frequencies. *Phys. Rev.* **1946**, *69*, 681.
3. Kleppner, D. Inhibited spontaneous emission. *Phys. Rev. Lett.***1981**, *47*, 233
4. Swathi R.S.; Sebastian, K.L. Long range resonance energy transfer from a dye molecule to graphene has (distance)$^{-4}$ dependence. *J. Chem. Phys.* **2009**, *130*, 086101, doi:10.1063/1.3077292
5. Yariv, A.; Yeh, P. *Optical Waves in Crystals*; John Wiley: Hoboken, NJ, USA, 1984.

© 2020 by the authors. Submitted for possible open access publication under the terms and conditions of the Creative Commons Attribution (CC BY) license (http://creativecommons.org/licenses/by/4.0/).
